# Valproate-Induced Hyperammonemic Encephalopathy Successfully Treated With Levocarnitine in an Elderly Patient Without Liver Dysfunction

**DOI:** 10.7759/cureus.76603

**Published:** 2024-12-30

**Authors:** Masahiko Kaneko, Hisaharu Shikata, Hisafumi Kihara

**Affiliations:** 1 Department of Internal Medicine and Hematology, Uwajima City Hospital, Uwajima, JPN

**Keywords:** carnitine, encephalopathy, hyperammmonemia, levocarnitine, valproate

## Abstract

An 87-year-old male with a history of seizure disorder on long-term prophylaxis with valproate and concomitant levetiracetam presented with impaired consciousness and anorexia. The evaluation revealed a markedly elevated blood ammonia level of 518 μmol/L and decreased serum carnitine levels, leading to a diagnosis of valproate-induced hyperammonemic encephalopathy in the absence of liver dysfunction. Discontinuation of valproate, continuation of levetiracetam, and initiation of levocarnitine supplementation and branched-chain amino acid infusion resulted in a durable resolution of symptoms. This case underscores the importance of promptly considering valproate-induced hyperammonemic encephalopathy due to carnitine depletion in patients on long-term valproate therapy who develop acute encephalopathy and initiating levocarnitine treatment for effective management.

## Introduction

Valproate, introduced in the United States in 1978 as an anticonvulsant, is widely used to treat partial and generalized seizures, acute mania, and as prophylaxis for bipolar disorder and migraines. While generally safe, valproate is associated with hyperammonemia, an idiosyncratic adverse effect that is typically asymptomatic and does not require intervention [1]. The pathogenesis of valproate-associated hyperammonemia (VAH) is linked to the metabolites of valproate, which affect the urea cycle directly or indirectly. Furthermore, long-term or high-dose valproate therapy can exacerbate this risk by depleting serum carnitine, a crucial cofactor for mitochondrial β-oxidation [2]. Although VAH rarely progresses to clinical encephalopathy, it can, in rare cases, lead to valproate-induced hyperammonemic encephalopathy (VHE), a potentially life-threatening condition characterized by acute cognitive decline, lethargy, and neurological dysfunction [3]. VHE is more frequent when valproate is used in combination with other enzyme-inducing antiepileptic drugs. Although age is not a known risk factor for VAH [4], its role in the development of VHE in elderly patients requires further exploration.

We herein report a case of VHE in an elderly male patient on valproate and concomitant levetiracetam. This report underscores the clinical challenges of diagnosing VHE in elderly patients and highlights the importance of measuring serum ammonia and carnitine levels when unexplained gastrointestinal symptoms or impaired consciousness are observed. Prompt treatment, such as discontinuation of valproate and administration of levocarnitine, can lead to rapid symptom resolution and prevent severe complications.

## Case presentation

An 87-year-old man from a group home was admitted to the Department of Internal Medicine, Uwajima City Hospital with a one-day history of altered mental status, decreased oral intake, lethargy, and decreased muscle tone. He had a nine-year history of seizure disorder due to sequelae of cerebral infarction, which had been controlled with valproate 800 mg to 1200 mg. He had been receiving levetiracetam 1000 mg in addition to valproate 1200 mg since generalized tonic-clonic two years prior to the current presentation. He also had long-standing hypertension, hyperuricemia, and vasospastic angina, all of which were well-controlled with benidipine, febuxostat, and nicorandil, respectively. Furthermore, he was not taking any concomitant agents that could affect components of the hepatic cytochrome activity system until the onset of the current presentation. On arrival, he exhibited indifference and mild upper limb tremor and his Glasgow Coma Scale (GCS) score was 9 (E2, V2, M5). His vital signs included a body temperature of 36.3°C, pulse rate of 82 bpm with regular rhythm, blood pressure of 128/72 mmHg, respiration rate of 14 breaths/minute, and oxygen saturation of 99% (ambient air). On a neurological examination, his pupils were 3.5 mm with normal light reflex bilaterally, but horizontal nystagmus was slightly observed. There was normal deep tendon reflex, but no limb paralysis, paresthesia, and pathological reflex. The findings of other physical examinations were normal. The results of initial blood tests, including the white blood cell count, hemoglobin level, blood sugar, liver function test, clotting screen, arterial blood gases, serum levels of C-reactive protein (CRP), and thyroid function test, were within the normal ranges. However, the levels of serum lactate dehydrogenase (LDH, 239 U/L; normal range: 100-200 U/L) and creatinine (Cr, 1.09 mg/dL; normal range: 0.36-1.06 mg/dL) were slightly elevated. The platelet count of 13.3 × 104/µL (normal range: 15.0-45.0 × 104/µL) and serum potassium level at 3.5 mmol/L (normal range: 3.5-4.9 mmol/L) were slightly decreased. Serum tests for hepatitis B and C, and hyaluronic acid as well as autoimmune diseases gave negative results. These data are shown in Table 1. Electrocardiogram, chest X-ray, and non-contrast-enhanced head computed tomography (CT) demonstrated no abnormalities. Whole-body CT also demonstrated no abnormalities and no portosystemic shunt was detected. Head magnetic resonance imaging (MRI), which was performed because cerebral infarction or encephalitis could not be ruled out, revealed no abnormalities. Per a review of the literature, it had been reported that valproate can affect the urea cycle, leading to a buildup of the level of blood NH3. Therefore, there was concern that valproate was the inciting cause of this patient’s presentation. The blood concentration levels of NH3 and valproate were immediately tested for a differential diagnosis of consciousness disorder. The level of blood NH3 was found to be markedly elevated at 518 µg/dL (normal range: 30-86 µg/dL). However, the blood valproate level was only slightly elevated (106 µg/mL; therapeutic range: 50.0-100.0 µg/dL). Furthermore, the serum levels of total and free carnitine measured at that time were decreased at 29.6 µmol/L (normal range: 45-91 µmol/L) and 18.0 µmol/L (normal range: 36-74 µmol/L), respectively (Table 1).

**Table 1:** Laboratory findings on admission. WBC: white blood cells; RBC: red blood cells; Hb: hemoglobin; T-Bil: total bilirubin; AST: aspartate aminotransferase; ALT: alanine aminotransferase; LDH: lactate dehydrogenase; ALP: alkaline phosphatase; GGT: γ-glutamyltransferase; BUN: blood urea nitrogen; Cre: creatinine; Na: sodium; K: potassium; Cl: chloride; CPK: creatinine phosphokinase; CRP: C-reactive protein; HbA1c: glycosylated hemoglobin; TSH: thyroid stimulating hormone; HBsAg: hepatitis B surface antigen; HCV: hepatitis C virus; Ab: antibody; NH3: ammonia; PaCO_2_: partial pressure of carbon dioxide; PaO_2_: partial pressure of oxygen; HCO_3_: bicarbonate; O2: oxygen.

Parameters	Normal range	Patient results	Parameters	Normal range	Patient results
WBC	4.0–9.0 × 10^3^/μL	4.58	CRP	0.00–0.30 mg/dL	0.02
RBC	450–510 × 10^4^/μL	430	HbA1c	4.6–6.2%	5.1
Hb	12.0–16.0 g/dL	15.0	TSH	0.54–4.54 μU/mL	5.33
Platelets	15.0–45.0 × 10^4^/μ	13.3	fT3	2.20–4.30 pg/mL	1.68
T-Bil	0.2–1.2 mg/dL	0.6	HBsAg	(-)	(-)
AST	13–33 U/L	22	HCV-Ab	(-)	(-)
ALT	8–42 U/L	9	Vitamin B1	4.0–9.0 ng/mL	21
LDH	100–200 U/L	239	NH3	30–86 μg/dL	518
ALP	3.4–4.8 U/L	141	Valproic acid	50.0–100.0 μg/mL	106
GGT	11–58 U/L	32	Carnitine	45.0–91.0 μmol/L	29.6
BUN	5–23 mg/dL	10	Free carnitine	36.0–74.0 μmol/L	18.0
Cre	0.36–1.06 mg/dL	1.09	pH	7.35–7.45	7.49
Na	135–149 mmol/L	145	PaCO_2_	35–45 mmHg	35
K	3.5–4.9 mmol/L	3.5	PaO_2_	80–90 mmHg	105
Cl	96–108 mmol/L	106	HCO_3_	22–26 mmol/L	27
CPK	62–287 U/L	121	O2 saturation	92–96%	98

**Figure 1:**
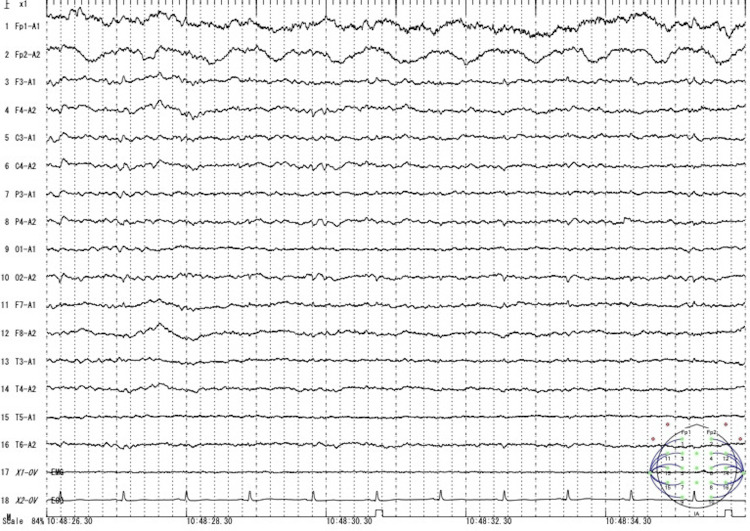
Electroencephalogram on day five of hospitalization. The electroencephalogram showed intermittent generalized slowing consistent with mild encephalopathy, but no electrographic seizures or interictal epileptiform activity were observed.

At this point, metabolic encephalopathy due to hyperammonemia was considered and he was started on intravenous branched-chain amino acids (BCAA)-containing fluid 500 mL once a day and oral levocarnitine (L-carnitine, active isoform of carnitine) supplementation 1500 mg per day. Valproate was immediately discontinued because the cause of the hyperammonemia was thought to be related to valproate. However, levetiracetam 1000 mg per day caused little enzyme induction/inhibition and few drug interactions [5], and was continued as a single anticonvulsant. On the second day of hospitalization, his blood ammonia (NH3) level had decreased to 104 µg/dL, but the GCS score remained at 11 (E3, V3, M5). On day three, his mental state gradually improved, and he was able to engage in basic conversation. As a result, the BCAA infusion was discontinued. By day four, his consciousness fully recovered to a GCS score of 15 (E4V5M6), and he was able to walk independently. On day five, an electroencephalogram was performed, which showed intermittent generalized slowing consistent with mild encephalopathy, but there were no electrographic seizures or interictal epileptiform activity (Figure 1).

The blood NH3 level on the same day had further decreased to 68 µg/dL, leading to the discontinuation of oral levocarnitine administration on the ninth day of hospitalization. The patient was eventually diagnosed with VHE. He responded well to the above-mentioned treatment and was discharged from the hospital without any sequela on post-admission day 10. At the outpatient visit one month after discharge, he had continued to take levetiracetam 1000 mg per day, and his blood NH3 level was within the normal range at 68 µg/dL. Additionally, the serum levels of total carnitine and free carnitine, which were measured simultaneously, were 46.1 µmol/L and 36.7 µmol/L, respectively, both within the normal range.

## Discussion

The present case of VHE, characterized by acute and progressive impaired consciousness and anorexia, concomitant levetiracetam, and carnitine deficiency, underscores several critical clinical insights. While hyperammonemia is a well-known idiosyncratic effect of valproate, symptomatic VHE is rare but potentially life-threatening, characterized by acute cognitive decline, gastrointestinal symptoms, and lethargy. This highlights the importance of maintaining a high index of suspicion for VHE in patients on long-term valproate therapy presenting with altered mental status, even in the absence of hepatic dysfunction.

VAH in the absence of liver dysfunction is a well-documented unique idiosyncratic reaction described in studies focusing on epilepsy and psychiatric patients [6]. The pathophysiological mechanisms for which valproate causes hyperammonemia are multifactorial, primarily involving carnitine depletion [7-9]. Carnitine is an essential cofactor that facilitates the transfer of fatty acids such as valproate into the mitochondria for β-oxidation in the liver [7]. Valproate reduces carnitine levels by inhibiting its reabsorption in the renal tubules and binding to form valproylcarnitine, which is excreted renally [8]. With long-term or high-dose therapy, valproate also reduces the renal tubular reabsorption of free carnitine, resulting in an increased risk of serum NH3 elevation [9]. Carnitine deficiency impairs the urea cycle, leading to decreased ammonia elimination and hyperammonemia. In addition, carnitine is primarily distributed in the muscles, but elderly patients, as in this case, generally have less muscle mass. Therefore, anorexia and drug-induced carnitine deficiency were more likely to occur, and it is possible that hyperammonemia worsened even without a high valproate concentration. Although it is well-documented that carnitine deficiency is linked to liver cirrhosis, VAH is distinct from hyperammonemia secondary to liver failure. One recognized predictor is the concomitant administration of hepatic cytochrome P450 (CYP) enzyme-inducing anticonvulsants, namely, phenytoin, phenobarbital, and carbamazepine, and newer-generation drugs such as topiramate. Enzyme-induced pharmacokinetic drug interactions are known to decrease serum valproate concentrations, requiring the administration of greater doses to maintain its narrow therapeutic index, which could have contributed to VAH [10]. However, the present case taking valproate had received the concomitant levetiracetam, a new-generation drug that has no CYP enzyme-inducing or inhibitory effects. Since a case of VHE with valproate monotherapy has been reported [11], it is considered that the VHE in this case was not affected by the concomitant levetiracetam. Encephalopathy due to hyperammonemia, which occurs in 0.8% to 2.52% of patients taking valproate [12,13], is a rare but life-threatening consequence, and its exact mechanism of action is currently not well understood. Unlike hyperammonemia secondary to liver dysfunction, VHE often occurs without significant liver enzyme abnormalities, necessitating a distinct diagnostic approach. In addition, in the results from most cohort studies to date, neither valproate dosage nor exposure duration were predictors, which highlights the idiosyncratic nature of this phenomenon [7,14,15]. Although symptoms can initially be gastrointestinal, with the patient presenting with anorexia and nausea, or behavioral, such as agitation, such clinical scenarios are commonplace among elderly patients with mild cognitive decline, and this drug-related complication can be easily overlooked [16,17]. Therefore, the potential differential diagnosis for VHE is broad and includes intracerebral hemorrhage, central nervous system infections, metabolic derangements, and other toxicities. The diagnosis of VHE is based on the history and suggestive clinical findings, and it is confirmed by an elevated serum NH3 concentration, which helps distinguish it from other conditions. Common causes of altered mental status, such as hypoglycemia and hyponatremia, should be ruled out during the initial evaluation with basic laboratory tests. In this way, recognition of VHE requires a high level of clinical suspicion by clinicians, as the clinical presentation is nonspecific and correlates poorly with dosage, blood levels, or duration of treatment [18]. This case emphasizes the need for prompt intervention. The adequate management of VHE is the immediate discontinuation of valproate, which has a success rate of 56.3% [19], and the initiation of treatment to lower serum NH3 levels, such as BCAA infusion and levocarnitine supplementation. Hemodialysis is reasonable to perform in clinically severe VHE, including coma, with valproate concentration >900 µg/mL, or in cases of coma or respiratory depression requiring mechanical ventilation [20]. In this case, immediate discontinuation of valproate, coupled with measures to reduce ammonia levels such as BCAA infusion and levocarnitine supplementation, resulted in rapid clinical improvement. Serum carnitine testing plays a critical role in identifying carnitine deficiency, which may guide preventative and therapeutic strategies in patients at risk of VHE. Despite the growing evidence supporting the use of levocarnitine in the management, particularly the treatment, of VHE, the evidence for the efficacy of carnitine supplementation in the prevention of VHE is based on case reports and retrospective studies, and no high-quality prospective studies exist. Further research is needed to better understand the mechanisms, risk factors, and optimal management of this rare but serious adverse effect of valproate therapy.

## Conclusions

Clinicians should monitor for signs of VHE in patients taking valproate, especially those with elevated serum NH3 levels and low carnitine levels. If such patients develop gastrointestinal symptoms, impaired consciousness, or acute cognitive decline, valproate administration should be discontinued immediately. Early intervention, such as carnitine supplementation, shown to be effective in treating, can help reduce ammonia levels and prevent serious complications.
